# Tor-Sch9 deficiency activates catabolism of the ketone body-like acetic acid to promote trehalose accumulation and longevity

**DOI:** 10.1111/acel.12202

**Published:** 2014-03-20

**Authors:** Jia Hu, Min Wei, Hamed Mirzaei, Federica Madia, Mario Amparo, Shawna Chagoury, Brian Kennedy, Valter D Longo

**Affiliations:** 1 Longevity Institute, Davis School of Gerontology, University of Southern CaliforniaLos Angeles, CA, 90089, USA; 2 Department of Biological Sciences, School of Dornsife College of Letters, Arts and Sciences, University of Southern CaliforniaLos Angeles, CA, 90089, USA; 3 DiBiMeF, Universita’ di Palermo90133, Palermo, Italy; 4 Buck Institute for Research on AgingNovato, CA, 94945, USA

**Keywords:** acetic acid, aging, chronological lifespan, leucine, Sch9

## Abstract

In mammals, extended periods of fasting leads to the accumulation of blood ketone bodies including acetoacetate. Here we show that similar to the conversion of leucine to acetoacetate in fasting mammals, starvation conditions induced ketone body-like acetic acid generation from leucine in *S. cerevisiae*. Whereas wild-type and *ras2Δ* cells accumulated acetic acid, long-lived *tor1Δ* and *sch9Δ* mutants rapidly depleted it through a mitochondrial acetate CoA transferase-dependent mechanism, which was essential for lifespan extension. The *sch9*Δ-dependent utilization of acetic acid also required coenzyme Q biosynthetic genes and promoted the accumulation of intracellular trehalose. These results indicate that Tor-Sch9 deficiency extends longevity by switching cells to an alternative metabolic mode, in which acetic acid can be utilized for the storage of stress resistance carbon sources. These effects are reminiscent of those described for ketone bodies in fasting mammals and raise the possibility that the lifespan extension caused by Tor-S6K inhibition may also involve analogous metabolic changes in higher eukaryotes.

## Introduction

The mechanisms underlying the aging process in mammals remain poorly understood. The budding yeast *Saccharomyces cerevisiae* has served as a primary model organism to study the mechanisms of aging. For instance, yeast has been responsible for the identification of two of the major pro-aging pathways: the Tor/S6K and the Ras/cAMP/PKA (Longo *et al*., 1996; Longo 1999; Pedruzzi *et al*., 2000, 2003; Fabrizio *et al*., 2001; Kaeberlein *et al*., 2005; Medvedik *et al*., 2007; Steffen *et al*., 2009; McCormick *et al*., 2011). *S. cerevisiae* has also provided some of the initial links between pro-aging pathways and age-dependent genomic instability (McMurray & Gottschling, 2004; Madia *et al*., 2009).

The yeast chronological lifespan (CLS), a measure of the chronological survival of a postmitotic population of cells, and the replicative lifespan (RLS), a measure of the replicative potential of an individual mother cell, have been the two major methods to assess yeast aging (Kaeberlein & Kennedy, 2005; Longo *et al*., 2012). Mutations in either Tor or Sch9, yeast orthologs of mammalian mTOR and S6K, respectively, and in the Ras/cAMP/PKA pathway extend both the CLS and RLS (Fabrizio *et al*., 2001; Fabrizio *et al*., 2003; Pan & Shadel, 2009; Laplante & Sabatini, 2012) in part by activating stress resistance serine/threonine kinase Rim15 and its downstream transcription factors Msn2/4 and Gis1 (Pedruzzi *et al*., 2000; Wei *et al*., 2008). Because the Tor/Sch9 pathway is known to be activated primarily by amino acids, while the Ras/cAMP/PKA pathway is turned on in large part by glucose and considering the key role of Rim15, Msn2/4, and Gis1 in the lifespan extension caused by calorie restriction (CR), these pathways represent the central pro-growth and pro-aging signaling network activated by nutrients (Longo & Finch, 2003; Wei *et al*., 2008). In fact, yeast cells are nonviable if they lack both Ras1 and Ras2 or Tor1 and Tor2 (Toda *et al*., 1985; Barbet *et al*., 1996).

Because high level of acetic acid promotes apoptosis in yeast, others have proposed that acetic acid and not ethanol is the primary factor promoting culture acidification, chronological aging, and apoptotic death (Burtner *et al*., 2009). It has also been proposed that CR increases chronological survival by reducing extracellular acetic acid and that the longevity of *sch9Δ* and *ras2Δ* mutants is due to reduced acetic acid production during the proliferative phase (*sch9Δ*) and acetic acid resistance after nutrient exhaustion (*sch9Δ* and *ras2Δ*) (Burtner *et al*., 2009). We instead provide strong evidence that, at physiological levels, acetic acid promotes aging simply by activating pro-aging pathways in yeast, analogously to glucose and ethanol.

In this study, we investigated the role of acetic acid in chronological aging and examined its source and impact on the lifespan of wild-type and long-lived *S. cerevisiae*.

## Results

### Yeast mutants deficient in the Ras/cAMP/PKA and Tor/Sch9 pathways exhibit different patterns of ethanol and acetic acid accumulation during chronological aging

**Figure 1:**
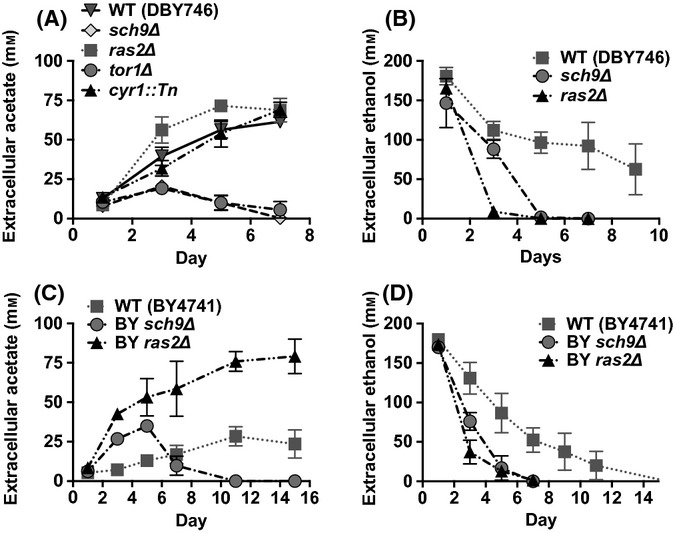
Quantification of extracellular acetate and ethanol during chronological aging extracellular acetate (A) and ethanol (B) concentrations of wild-type, *sch9∆, ras2∆, tor1∆,* and *cyr1*::Tn mutants in DBY746 background on days 1, 3, 5, and 7. Data are presented as mean ± SEM (*n* = 3–7). Extracellular acetate (C) and ethanol (D) concentrations of wild-type, *sch9∆, ras2∆* mutants in BY4741 background (*n* = 3–6).

To establish the relationship between carbon source metabolites and CLS, we measured the levels of ethanol and acetic acid in chronologically aged yeast cultures (Fig. 1). Wild-type cells accumulated acetate during chronological aging, but the deletion of either *TOR1* or *SCH9* resulted in depletion of extracellular acetate after a small peak on day 3 (Fig. 1A). These data indicate that the Tor/Sch9 pathway blocks the utilization of acetate. By contrast, mutations in *RAS2* or *CYR1,* the central components of the other yeast pro-aging pathway with orthologs that promote aging in mice (Ferbeyre *et al*., 2002; Yan *et al*., 2007), accumulated similar or even higher levels of acetate compared to the wild-type strain (Fig. 1A). These results suggest that during the early stages of lifespan, as extracellular glucose and other nutrients become depleted, the absence of Tor/Sch9 signaling promotes a metabolic change that utilizes acetic acid, analogous to the generation and use of the ketone bodies, acetoacetic acid and β-hydroxybutyrate, by mammalian cells during fasting (Hawkins *et al*., 1986; Cahill, 2006).

Calorie restriction is well known to extend longevity in a wide range of organisms, including yeast (Kennedy *et al*., 2007; Anderson & Weindruch, 2010). As we had previously shown (Fabrizio *et al*., 2005), the ethanol content remains relatively high in wild-type cultures until day 9, while it drops by day 3 in *sch9*Δ and *ras2Δ* mutants (Fig. 1B). These data rule out the possibility that wild-type cells die from carbon source starvation. Thus, the removal of ethanol may partially contribute to the lifespan extension effects of Sch9 or Ras2 deficiency. To test whether these effects of Tor/Sch9 and Ras/cAMP/PKA on extracellular carbon sources are common characteristics of yeast cells, we also measured ethanol and acetate production in the BY4741 strains, one of the most widely studied yeast genetic background, and obtained similar results (Fig. 1C,D). However, early carbon source depletion may only explain part of the lifespan extension in specific long-lived mutants, because acetic acid remained at very high levels in *ras2Δ* cultures (Fig. 1A,C).

### Ethanol, acetate, and acidification promote aging by reducing the activity of stress-response transcription factors

**Figure 2:**
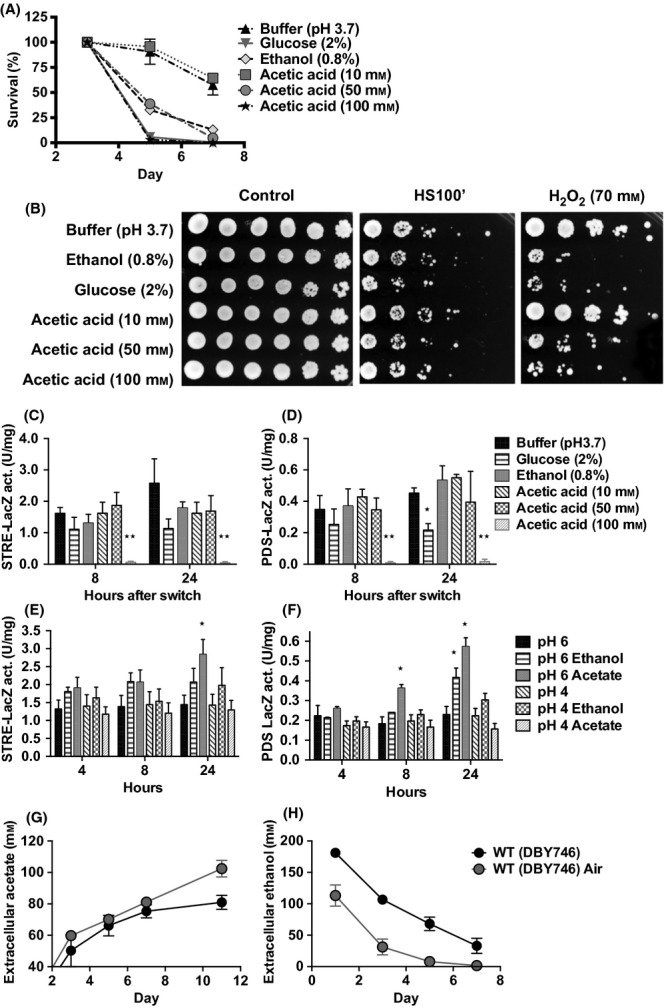
Effect of carbon sources on chronological aging and stress response. (A) Chronological survival of wild-type (DBY746) cells in different media. Day 3 cells were washed and transferred to MES buffer in the presence of different carbon sources at the indicated concentrations. All media were adjusted to the same pH as the control (buffer only). (B) Stress resistance assay of wild-type strain (DBY746) 24 h after the switch to various buffers. Cells were heated for 100 min at 55 °C or treated with 70 mm H_2_O_2_ for 30 min. A representative experiment was shown. (C, D) LacZ reporter assay. Wild-type cells with chromosomally integrated STRE- or PDS-LacZ reporter genes were switched to buffer containing different carbon sources after three days of growth in standard SDC medium. The STRE-LacZ (C) and PDS-LacZ (D) activities were measured 8 h and 24 h after the switch. Data shown are mean ± SEM (*n* = 3–4). (E, F) Wild-type (DBY746) cells were transferred to 40 mm MES buffer (pH 6, pH 4) supplemented with ethanol (0.8%) or acetic acid (50 mm) after two days of growth in SDC medium. The STRE-LacZ (E) and PDS-LacZ (F) activities were measured 4, 8, and 24 h after the switch (*n* = 3). Star denotes *P* < 0.05; double stars denote *P* < 0.01. (G, H) Wild-type cells were grown in SDC medium under the standard condition with limited aeration (aluminum caps) or high aeration condition (loose plastic caps). Extracellular acetate and ethanol concentrations were measured every other day.

To better understand how carbon sources affect CLS, we switched day 3 cells to MES buffer containing glucose, ethanol, or acetic acid (Fig. 2). As shown in Fig. 1, the average extracellular acetate concentration between day 3 and day 7, the period during which over 50% of the cells died, was approximately 50 mm. The pH of the buffer was adjusted to that reached by wild-type cells in the standard CLS assay (pH 3.7). The standard 2% glucose concentration or a 100 mm acetic acid concentration (twice the physiological levels) caused the highest mortality in agreement with previous reports (Granot & Snyder, 1991; Ludovico *et al*., 2001; Burtner *et al*., 2009). By contrast, a physiological concentration of ethanol (0.8%) or of acetic acid (50 mm) using the standard low aeration protocol (Longo *et al*., 2012) did not shorten lifespan to the same level as 2% glucose or 100 mm acetic acid (Fig. 2A). This observation suggests that at physiological levels, acetic acid affects chronological aging at a level similar to that of ethanol, but lower than that of glucose (Wei *et al*., 2009). To examine whether the carbon source influences stress resistance, we challenged cells with heat shock and oxidative stress (H_2_O_2_ treatment for 30 min) after switching them to various carbon sources for 24 h. At physiological concentrations, all the carbon sources sensitized yeast cells only to oxidative stress (Fig. 2B).

Gis1 and Msn2/4 are stress-response transcription factors required for lifespan extension of Tor/Sch9- or Ras/cAMP/PKA-deficient cells. To further understand how carbon sources affect the stress response, we employed STRE- and PDS-driven LacZ reporter gene assays to monitor Msn2/4 and Gis1 transactivation, respectively (Fig. 2C,D). High concentration of acetic acid (100 mm) completely repressed the STRE- and PDS-dependent transactivation, while its physiological concentrations, up to 50 mm, reduced the activation of these transcription factors (Fig. 2C,D). This effect could explain the cytotoxicity of super-physiological levels of acetic acid. Cells in glucose medium showed a significant reduction in PDS-driven gene expression in agreement with the stress resistance results (Fig. 2B,D). Based on these results, we conclude that physiological concentration of either acetic acid or ethanol in combination with media acidification similarly affects cellular protection and survival.

To determine the relative contribution of acidification to cellular survival and stress sensitization, we monitored the effect of pH on the activity of the Msn2/4 and Gis1 stress resistance transcription factors (Fig. 2E,F). Similar to the experiment above, we switched cells to MES buffer (pH 6.0 or 4.0) supplemented with either physiological level of ethanol (0.8%) or acetate (50 mm). Mild acidification (pH 4.0) did not significantly alter PDS- or STRE-dependent transactivation. In the presence of acetic acid, the increase in pH from 4 to 6 elevated STRE- and PDS-dependent LacZ activity (Fig. 2E,F). This observation may explain why buffering the acidic media to pH 6 extends longevity (Fabrizio *et al*., 2005; Burtner *et al*., 2009; Kaeberlein, 2010). By contrast, a pH increase promoted PDS- but not STRE-dependent LacZ activity in ethanol medium (Fig. 2E,F). We also buffered the spent medium collected from day 3 wild-type cultures to pH 6.0 and found that PDS-LacZ, but not STRE-LacZ, activity was elevated and reached an almost two-fold increase compared to cells in the unbuffered medium by 48 h (Figure S1A,B Supporting Information). These results suggest that Gis1 transactivation via the PDS elements, which are mostly negatively regulated by the Tor/Sch9 signaling pathway (Wei *et al*., 2008), may be responsible for part of the effects of medium acidification and ethanol or acetic acid on stress resistance and CLS.

In order to reconcile these data and the proposal by others that acetic acid is a toxic molecule that causes acute cytotoxicity (Kaeberlein, 2010; Longo *et al*., 2012), we investigated whether differences in technical procedures may be responsible for the different results. Using loose-fitting plastic caps that allow high aeration instead of the aluminum foil caps that limit aeration used in our method, Burtner *et al*. (2009) showed that ethanol was depleted rapidly and acetic acid accumulates in aging yeast cultures. We performed our viability assay using both methods. The evaporation rate was much higher in plastic cap-covered flasks as the volume decreased by 50–70% by day 11 compared to a 15% decrease in flasks with aluminum foil caps (data not shown). The higher aeration also caused early depletion of ethanol and caused the levels of acetic acid to reach a concentration close to 100 mm (Fig. 2G,H). The pH of wild-type cultures was maintained between 3.5 and 3.8 throughout the lifespan using both methods (Figure S1C Supporting Information). These results indicate that the methodology used by Burtner *et al*. caused early depletion of ethanol and increased the levels of acetic acid to a toxic range, in contrast to our method in which ethanol remains the major carbon source and the concentration of acetic acid reached between days 3 and 7 is approximately 50–60 mm. This demonstrates that CLS, similar to aging assays in other organisms, is dependent on environmental conditions.

### Mutations that reduce acetic acid levels do not affect longevity or acidification

**Figure 3:**
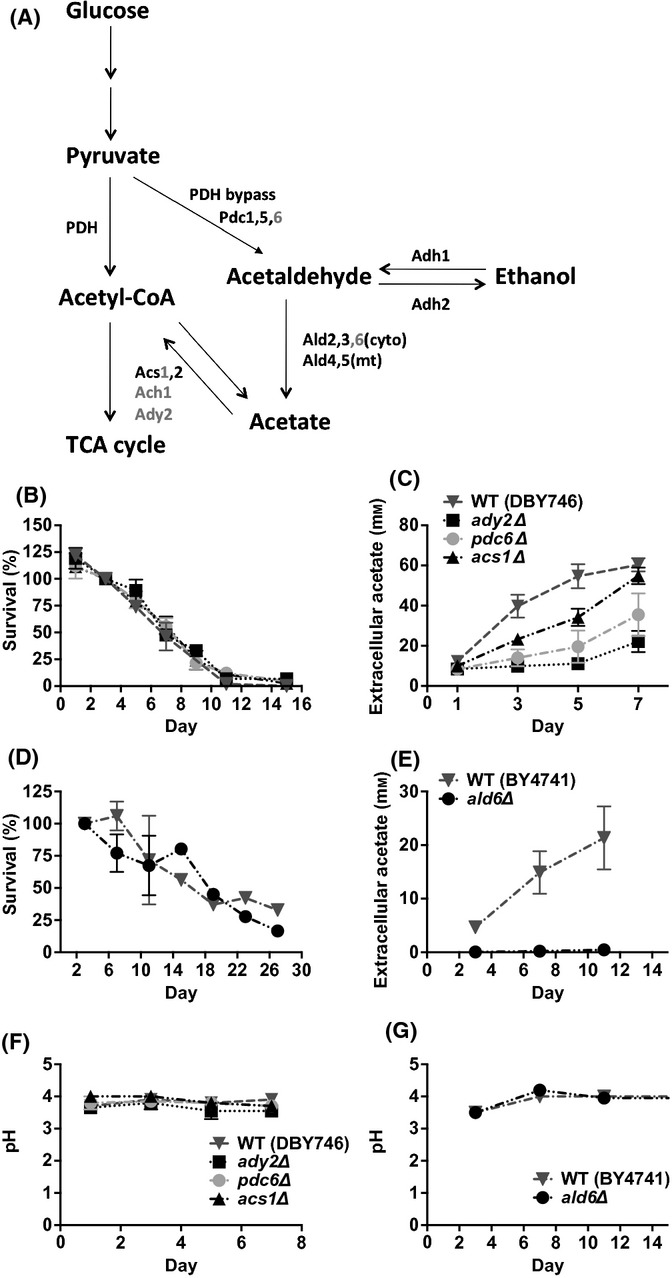
Genetic mutations effecting acetic acid levels do not alter, chronological lifespan, and pH. (A) The glucose metabolism pathway leading to ethanol and acetate generation. Chronological survival (B) and extracellular acetic acid concentration (C) of wild-type (DBY746), *ady2∆*, *pdc6∆*, and *acs1∆* mutants (B, *n* = 5–9; C, *n* = 3–7).Chronological survival (D) and extracellular acetic acid concentration (E) of wild-type (BY4741) and *ald6∆* mutants (*n* = 3). The pH of conditioned media during chronological aging of DBY746 (F), BY4741 (G), wild-type cells and their derivatives.

To further investigate the role of acetic acid in *S. cerevisiae* chronological aging, we compared the survival of isogenic yeast strains carrying mutations in genes involved in acetic acid metabolism (Fig. 3A). *ADY2* encodes an acetate transporter, and its disruption has been reported to affect acetic acid cellular uptake (Paiva *et al*., 2004). *PDC6* encodes an isoform of the pyruvate decarboxylase, which decarboxylates pyruvate to acetaldehyde. Deletion of *PDC6* is expected to affect the production of acetaldehyde from pyruvate and, in turn, reduce acetate production (Pronk *et al*., 1996). *ACS1* is an acetyl-coA synthetase, whose deficiency can affect acetate production (De Virgilio *et al*., 1992). Although mutants lacking Ady2, Pdc6, or Acs1 caused major reductions in the levels of acetic acid (Fig. 3C), none of them displayed increased CLS or variation in ethanol concentration or pH (Fig. 3B,F, Figure S1D Supporting Information), suggesting that the levels of extracellular acetic acid using the standard CLS method used in our laboratory do not play a major role in either CLS lifespan or medium acidification. We confirmed our results in the BY4741 mutants lacking *ALD6*, a member of the acetaldehyde dehydrogenase (ACDH) family that catalyzes conversion of acetaldehyde into acetic acid (Fig. 3A). Although little acetate was detectable in *ald6*∆ culture during chronological aging (Fig. 3E), the chronological lifespan and medium pH did not differ significantly from those of the wild-type cells (Fig. 3D,G). Although unlikely, we cannot rule out that these mutants may have common defects, other than those related to acetic acid accumulation, which prevent lifespan extension. Furthermore, it is possible that aeration condition may influence the role of these genes and pathways on acidification.

### Acetic acid is a ketone body-like metabolite generated in part from leucine metabolism

**Figure 4:**
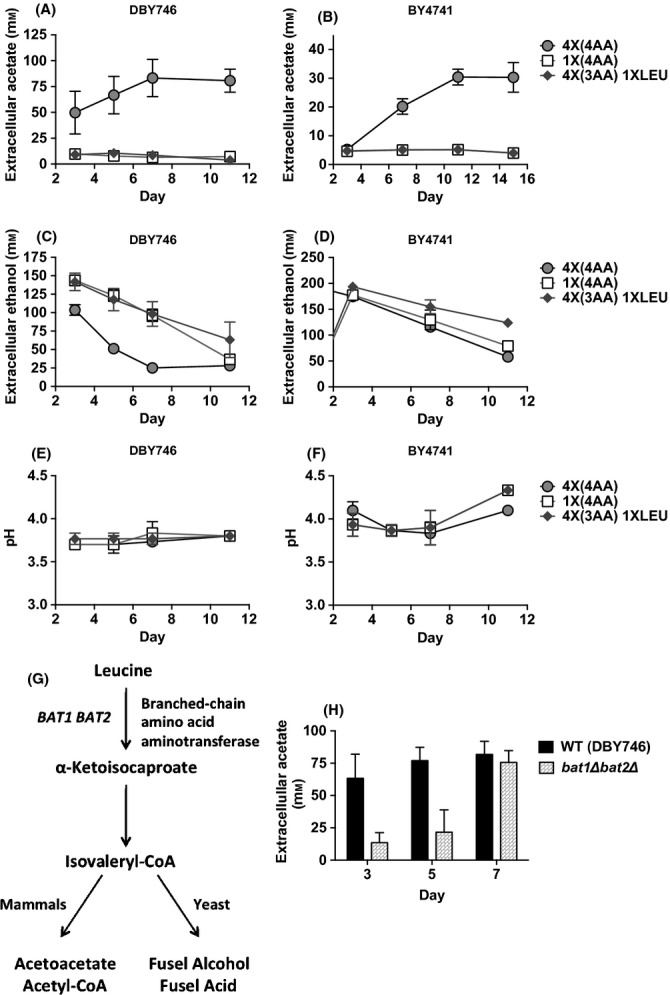
Branched chain amino acid leucine affects acetic acid generation. Wild-type cells (DBY746 and BY4741) growing in SDC medium supplemented with 4X (4X 4AA) or 1X HIS, URA, LEU, TRP (1X 4AA) or 1X LEU with 4XHIS, URA, TRP (4X 3AA 1X LEU). (A, B) Extracellular acetate concentration. (C, D) Extracellular ethanol concentration (*n* = 3–5). (E, F) pH of the cultures during chronological aging. (G) Leucine catabolism pathways in yeast and mammals. (H) Extracellular acetate concentration in cultures of wild-type cells and of mutants lacking *BAT1* and *BAT2* genes (*n* = 6); *P < 0.05, paired t-test, *bat1Δ bat2Δ* compared to WT.

In mammalian cells, ketone bodies, including acetone, acetoacetic acid, and β-hydroxybutyric acid, are generated from fatty acids and ketogenic amino acids catabolism. We hypothesized that acetic acid is a ketone body-like metabolite that serves as an energy source during periods of glucose depletion. We tested whether leucine could affect acetic acid levels. The commonly used laboratory yeast strains contain mutations in amino acid biosynthetic genes (see experimental procedures). Standard growth medium is supplemented with excess 4X amino acids (leucine, histidine, and uracil, in both backgrounds and tryptophan only for DBY746) to compensate for these genetic deficiencies (Fabrizio *et al*., 2001). When we reduced the excess amino acids or only leucine to 1X, the extracellular acetate concentration was markedly reduced, while ethanol levels were not affected (Fig. 4A,C). Notably, cell density of the 1X leucine culture reached only 25–30% of that reached by growth in 4X leucine (standard medium), but the acetic acid production remained greatly reduced when the effects were normalized per cell number (Figure S2A,B Supporting Information). Moreover, we evaluated CLS of DBY746 wild-type cells in 1X leucine media and observed that lifespan was reduced when compared to same cells grown in standard media (Figure S4A Supporting Information). Similar results were obtained with the BY4741 genetic background (Fig. 4B,D). Furthermore, the major difference in acetic acid production did not cause significant differences in culture pH, indicating that acetic acid is not a major factor in medium acidification (Fig. 4E,F). An increase in leucine levels could promote acetate production in the BY4741 (Figure S2C Supporting Information), but not in the DBY746 wild-type strain, whose extracellular acetate levels were already high (data not shown).

Bat1 and Bat2 are the mitochondria and cytosolic branched-chain amino acid (BCAA) aminotransferases (Fig. 4G) in the leucine metabolism pathway (Dickinson, 2000). Deletion of either of these two genes had little effect on acetate accumulation, but the double knockout mutants showed reduced extracellular acetate levels during early chronological aging (Fig. 4H). However, chronological survival of the *bat1Δ bat2Δ* double mutants in the standard SDC did not differ significantly from those of wild-type cells in same media (Figure S4B Supporting Information). This observation is possibly due to utilization of an alternative pathway in the *bat1Δ bat2Δ* double mutants. These data suggest that acetate production shares similarities to that of ketone bodies generation by leucine in mammals.

To examine whether prototrophy and auxotrophy may affect the survival of the long-lived mutants, we introduced the *LEU2* gene in the *tor1Δ* and *sch9Δ* strains utilized in this study. As shown in Supplemental Figure S2D, both *tor1Δ* and *sch9Δ* mutants exhibit a longer lifespan in comparison with wild-type, with *sch9Δ* presenting a more pronounced extended CLS than *tor1Δ*.

### The acetate utilization by the *sch9*∆ mutant is dependent on Ach1

**Figure 5:**
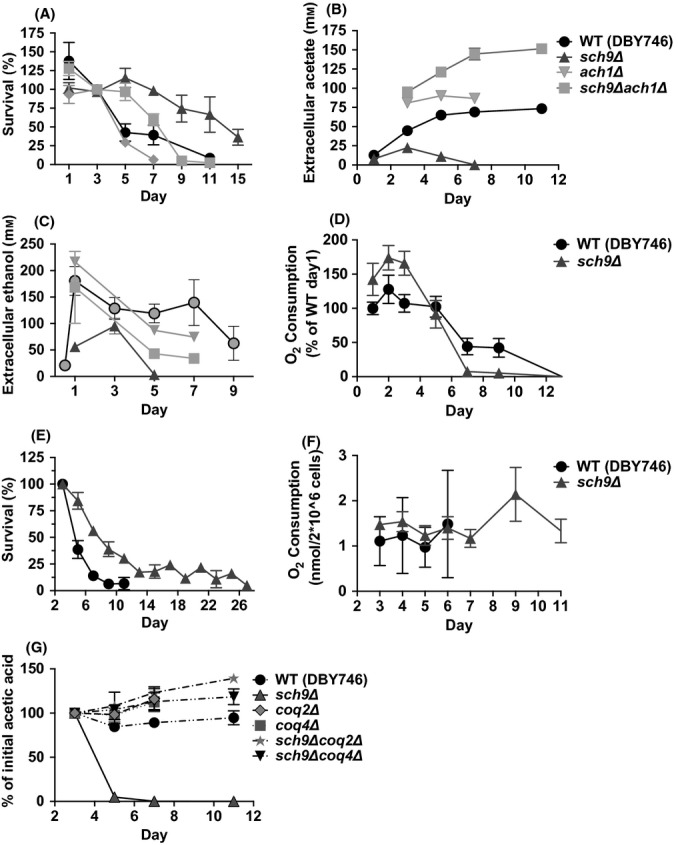
Ach1 and electron transport activities are important for acetic acid utilization. (A) Chronological survival of wild-type, *ach1∆, sch9∆*, and *sch9∆ach1∆* mutants (*n* = 7). (B, C) Extracellular acetic acid and ethanol concentrations during chronological aging (*n* = 4). (D) O_2_ consumption of wild-type (DBY746) and *sch9Δ* mutants during chronological aging. Data are presented as mean ± SE (*n* = 8–9). (E, F) WT (DBY746) and *sch9Δ* mutants were grown in SDC and switched to the carbon source mix medium (40 mm MES buffer, pH 3.7: with 50 mm acetic acid and 141 mm ethanol). The medium was changed on day 3 and replaced every other day. Chronological survival and O_2_ consumption were monitored for the entire chronological lifespan study. Data are presented as mean ± SEM (*n* = 7). (G) WT and deletion mutants were switched to 40 mm MES buffer (pH 3.7) containing 25 mm acetic acid. Acetate utilization was monitored.

The pro-longevity effect of Tor/S6K inhibition, demonstrated in yeast, worms, flies, and mice, is one of the most promising pharmacological interventions with the potential to extend healthy lifespan in humans (Fabrizio *et al*., 2001; Hansen *et al*., 2007; Harrison *et al*., 2009; Pan & Shadel, 2009; Katewa & Kapahi, 2011). To investigate the effect of Tor/Sch9 deficiency on acetic acid utilization, we studied the role of Ach1, a mitochondrial CoA transferase that catalyzes the CoA-SH transfer from succinyl-CoA to acetate with minor acetyl-CoA-hydrolase activity (Fig. 3A) (Fleck & Brock, 2009). The *ACH1* deletion mutants and wild-type cells displayed a similar mean lifespan, although the maximum lifespan of *ach1Δ* was shorter (Fig. 5A). The *sch9*∆ *ach1*∆ double mutations caused a reversal of the longevity effect of *sch9*Δ and led to a significant increase in ethanol and acetate accumulation compared to *sch9*∆ mutants (Fig. 5A–C). The acetate in the *sch9Δ ach1Δ* culture reached a concentration of over 100 mm (Fig. 2A). These results indicate that the activation of acetic acid utilization in cells deficient in Tor/Sch9 activity depends on *ACH1,* whose activity is required for lifespan extension in *sch9*Δ.

### Levels of nonfermentable carbon sources match respiration pattern in *sch9*∆ mutants

Down-regulation of the Tor1-Sch9 signaling pathway has been shown to induce an early increase in respiration rates (Bonawitz *et al*., 2007; Wei *et al*., 2009; Pan *et al*., 2011) as well as an early depletion in ethanol and acetic acid during chronological aging (Fig. 1A,B). We hypothesized that inhibition of Tor/Sch9 signaling increases respiration by promoting the catabolism of ethanol and acetic acid. Oxygen consumption measurements revealed that Sch9 deficiency induced a high rate of respiration at early stages of survival, but low respiratory rates by day 7 (Fig. 5D). In the BY4741 genetic background, deletion of *SCH9* exerted a similar but delayed effect on O_2_ consumption (Figure S3C Supporting Information). These metabolic changes matched the pattern of ethanol and acetic acid depletion during chronological aging. However, *sch9*∆ mutants remained long-lived when ethanol and acetic acid were replenished every other day at physiological levels (141 and 50 mm, respectively) during the CLS (Fig. 5E). The *sch9∆* mutants maintained a high metabolism (Fig. 5F), indicating that the effects of Tor/Sch9 deficiency on CLS are not simply due to entry into a low metabolism state. To examine the effect of each carbon source on respiration, we switched wild-type and *sch9∆* cells to MES buffer containing 0.8% (174 mm) ethanol or 50 mm acetic acid on day 3 and measured oxygen consumption 3 and 24 h after the switch (Figure S3A,B Supporting Information). Whereas ethanol promoted cellular respiration, acetic acid, which was also rapidly depleted by *sch9*∆ mutants, did not support high respiration even at points in which its levels were high and it was still detectable 24 h after the switch (data not shown, Figure S3A,B Supporting Information). Therefore, deficiencies in the Tor/Sch9 pathways activate an alternative metabolic mode that promotes rapid catabolism of ethanol, which induces high respiratory rates and a rapid catabolism of acetic acid.

To understand how long-lived mutants utilize acetic acid, we treated wild-type and *sch9∆* mutant cells with NaCN (0.25 mm), a respiration inhibitor, on day 1 or day 3. Treatment of *sch9*∆ mutants with NaCN every other day until day 7 delayed the utilization of acetic acid (Figure S3D Supporting Information). We also employed mutants with impaired respiratory function. Coq genes catalyze ubiquinone (coenzyme Q) biosynthesis, which serves to transport electrons between the respiratory enzyme complexes I, II, and III in the mitochondrial inner membrane. Deletion of *COQ2* or *COQ4* genes in wild-type cells and *sch9*∆ mutants led to impaired ethanol consumption and low acetic acid accumulation during chronological aging (Figure S3E,F Supporting Information). These results indicate that acetic acid catabolism in *sch9*∆ requires a fully functional respiratory chain, but does not necessarily result in elevated oxygen consumption in the absence of ethanol. Thus, Tor/Sch9 deficiency activates an alternative metabolic acetic acid catabolism mode that does not require high oxygen consumption.

### Deletion of *SCH9* promotes storage of energy reserves

**Figure 6:**
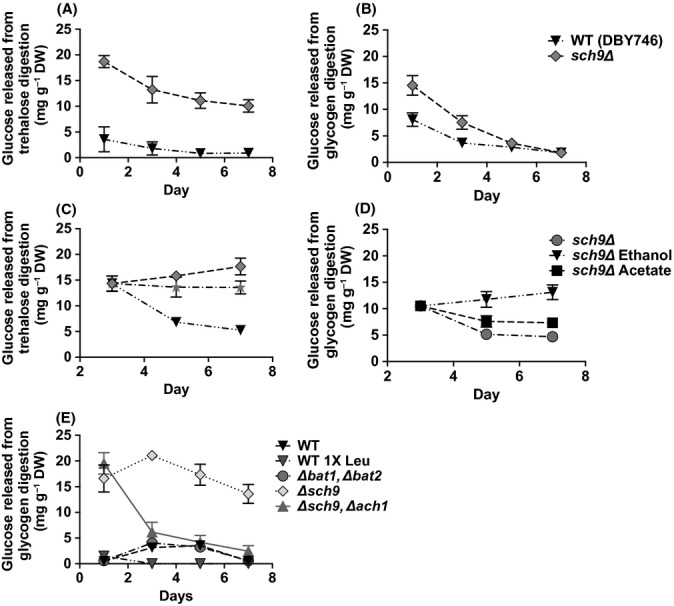
Acetic acid and reserve carbon sources. (A, B) Trehalose and glycogen contents were measured during chronological aging. (C, D) The *sch9Δ* mutant cells were switched to MES buffer (pH 3.7) containing 25 mm acetate or 0.8% ethanol on day 3. Trehalose and glycogen were measured. Measurements were normalized to cell pellet weight. (E) Trehalose was measured during chronological aging.

To determine whether acetic acid may contribute to other cellular functions not related to energy production, we measured stored nutrients. Storage carbohydrates, mainly glycogen and trehalose, serve as energy sources during stationary phase in yeast. Mutants unable to store or utilize the storage carbohydrates have significantly shortened CLS (Favre *et al*., 2008). We measured the glycogen and trehalose contents of cells aging chronologically. The levels of trehalose and glycogen decreased with age and reached very low levels in wild-type cells but were not depleted, even in very old organisms (Fig. 6). The *sch9*∆ mutants stored more than 2-fold higher glycogen and trehalose levels compared to wild-type cells on day 1 (Fig. 6A,B). A positive correlation between carbohydrate storage and cell survival has been shown previously (Ocampo *et al*., 2012). Glycogen does not appear to be as important because by day 5 wild-type cells and *sch9*∆ reached similar levels of this reserve nutrient (Fig. 6B). This suggests that the high level of trehalose may contribute to cellular protection in *tor1*∆ and *sch9*∆ mutants. We then treated cells with acetic acid or ethanol to determine whether these carbon sources can increase the accumulation of reserve carbohydrates. Interestingly, ethanol and acetic acid had different effects on the storage of carbon reserves. In *sch9*∆ mutants, acetic acid increased the accumulation of trehalose, while ethanol promoted its utilization. In contrast, ethanol, but not acetic acid, increased glycogen content (Fig. 6C,D). Therefore, ethanol and acetic acid trigger different metabolic pathways leading, respectively, to respiration and glycogen or trehalose storage.

Because Ach1 seems to be required for the gluconeogic conversion of acetic acid to trehalose, we evaluated intracellular concentration of trehalose in *sch9Δ, ach1Δ* double knockout cells and, as predicted, its levels are significantly reduced in comparison to those in *sch9Δ* mutants (Fig. 6E). To further strengthen our observations, we evaluated trehalose concentration in chronologically aged wild-type cells that are limited in leucine, as well as *bat1Δ bat2Δ* double mutant cells incubated in standard SDC. As hypothesized, wild-type cells cultured in 1X leucine had a lower accumulation of trehalose than that measured for cells in SDC media (Fig. 6E). Evaluation of chronologically aged *bat1Δ bat2Δ* mutant cells indicated that the intracellular accumulation of trehalose is not significantly different from that of the wild-type strain. We postulate that this could be in part due to pathways that are activated in the Bat1-, Bat2-deficient cells (Fig. 6E).

## Discussion

The effect of energy intake on lifespan is well documented in organisms ranging from bacteria to mice and other mammals. In fact, a 20–40% restriction in calories intake extends lifespan and reduces the incidence of a variety of diseases (Wei *et al*., 2008; Fontana *et al*., 2010). Because high levels of acetic acid have been shown to cause apoptosis in yeast (Ludovico *et al*., 2001), the observation that acetic acid accumulates in cultures of nondividing yeast led to the hypothesis that acetic acid plays a key role in yeast chronological aging (Burtner *et al*., 2009; Kaeberlein, 2010). Here, we show that under the high aeration conditions, ethanol is depleted and acetic acid accumulates reaching the toxic range associated with acute cell death, whereas under the standard aeration conditions used in our laboratory, ethanol and acetic acid represent the major carbon sources in the medium, with ethanol being the major pro-aging macronutrient. We also show that, analogously to the ketone bodies in mammals, acetic acid accumulates during the early periods of glucose depletion and can be generated from leucine catabolism in yeast cultures. Whereas wild-type cells and long-lived Ras pathway-deficient strains accumulate high levels of acetic acid, Tor/Sch9-deficient mutants rapidly catabolize it by an ACH1- and electron transport-dependent process that is required for lifespan extension and result in the storage of the protective reserve carbohydrate trehalose.

Relatively small differences in the methods used to measure lifespan may have pronounced effects on the rate of aging and cell death as well as the interpretation of the results (Gems & Partridge, 2013). We have shown that glucose and ethanol promoted aging and that medium acidification accelerated this process (Fabrizio *et al*., 2005). Furthermore, the calorie-restricted condition achieved by removing ethanol was sufficient to extend CLS (Fabrizio *et al*., 2005; Wei *et al*., 2009), analogously to the effect of reduction in glucose levels on both RLS and CLS. Burtner *et al*., on the contrary, proposed that it was the accumulation of acetic acid and not ethanol that limited yeast longevity and proposed that acetic acid may limit CLS not by promoting aging but by causing acute toxicity (Burtner *et al*., 2009; Kaeberlein, 2010). This difference, which appears to arise in part from the high aeration generated by experimental conditions adopted by Burtner *et al*., appears to be the result of an early depletion of ethanol and the accumulation of levels of acetic acid associated with cytotoxicity. Thus, to identify genes and pathways that are in fact affecting aging, it is important to use multiple experimental approaches that eliminate artifacts that may not directly impact aging (Longo *et al*., 2012).

Ketone bodies are the by-products of fatty acid catabolism in the liver and serve as a major source of energy during periods of food deprivation. The level of ketone bodies, normally very low in the plasma, increases when the glycogen becomes depleted and blood glucose level is low (Cahill, 2006). In yeast, acetate is produced by glucose metabolism, converted from acetaldehyde and acetyl-CoA. Here, we describe a number of parallels between yeast acetic acid and mammalian ketone bodies: (i) they are generated at high levels during periods of external glucose and glycogen depletion, (ii) leucine catabolism promotes the accumulation of ketone bodies in mammals and acetate in yeast (Bixel & Hamprecht, 1995), (iii) as for ketone bodies, yeast acetate can be transformed into acetyl-CoA which enters the TCA cycle for energy generation. The leucine metabolism pathway in yeast remains elusive. One previous study of leucine catabolism in yeast did not find acetate as a by-product, possibly because the experiments were not performed under glucose-limited conditions (Dickinson, 2000).

Sch9 and Tor1 deficiencies have been shown to extend lifespan, increase stress resistance, and promote genomic stability in *S. cerevisiae* (Fabrizio *et al*., 2001; Wei *et al*., 2009). Here, we report that deletion of *SCH9* causes acetate utilization in an *ACH1-* and electron transport chain-dependent manner. However, in *sch9Δ* mutants, acetic acid did not increase respiration as ethanol did, suggesting that Tor-S6K deficiency leads to a metabolic switch in which cells are able to utilize a ketone body-like metabolite for a respiration-independent purpose. Although it is not clear whether acetic acid utilization can result in ATP generation, we show that it promotes the accumulation of the stress resistance-related reserve carbon source trehalose, probably by a mechanism that requires an intact electron transport, as indicated by others (Filipak *et al*., 1992). In fact, it has been shown that petite cells lacking mitochondrial DNA stopped synthesizing trehalose as soon as exogenous glucose was consumed, while wild-type cells continued to accumulate the disaccharide (Enjalbert *et al*., 2000). A recent study suggests that the respiration thresholds are crucial for the extended CLS caused by CR and that trehalose was sufficient to restore normal CLS in respiration-deficient cells (Ocampo *et al*., 2012). Consistent with findings by others (Bonawitz *et al*., 2007; Pan *et al*., 2011), we demonstrate that down-regulation of the Tor/Sch9 signaling pathway induces an early increase in respiration rates during CLS and show that this is accompanied by early depletion of ethanol and acetic acid (Fig. 1A,B) as well as storage of trehalose (Fig. 6). These observations are in agreement with the findings that the effect of reduced TORC1 activity on life span in yeast may require an increased generation of ROS during growth phase. Thus, deficiency in Tor/Sch9 signaling may promote trehalose accumulation as part of a metabolic change that allows cells to catabolize acetic acid and survive under hypoxia or anoxia.

In agreement with our results, an early increase in respiration has been shown for Tor-deficient mutants and has been proposed to be required for lifespan extension (Bonawitz *et al*., 2007; Pan *et al*., 2011).

Here, we show that this early increase in respiration in Tor/Sch9-deficient cells is followed by a decrease in respiration after ethanol and acetic acid are largely depleted, indicating that the increased metabolism is reflecting the need to rapidly deplete extracellular carbon sources. However, carbon source depletion and the following hypometabolism do not fully explain the effects of Tor/Sch9 deficiency on lifespan, because *tor1*∆ and *sch9*∆ mutants display extended lifespan when carbon sources and respiration are maintained high and constant (Fig. 5E) (Wei *et al*., 2008). Similar to our findings, it has been reported that CR results in a 30% higher respiration rate in the growth phase and a decrease in respiration in the postdiauxic shift/stationary phase (Ocampo *et al*., 2012). This finding suggests that CR and reduced Tor/Sch9 signaling may regulate common genes, which first promote respiration and depletion of nonfermentable carbon sources and then cause a hypometabolic mode associated with lifespan extension (Wei *et al*., 2009). In agreement with protective effects of β-Hydroxybutyrate demonstrated in mammals (Shimazu *et al*., 2013)

In summary, our study suggests that acetic acid may represent a ketone body-like carbon source generated during periods of starvation and utilized by Tor/Sch9-deficient cells through an Ach1- and electron transport-dependent pathway which promotes the accumulation of the protective carbohydrate trehalose and lifespan extension.

## Experimental procedures

### Yeast strains

Most of the *Saccharomyces cerevisiae* strains used in this study were derived from DBY746 (*MAT*α*, leu2*-*3, 112, his3*∆*1, trp1*-*289, ura3*-*52, GAL+*). Some experiments were also confirmed in the BY4741 (*MATa, his3*∆*1, leu2*∆*0, met15*∆*0, ura3*∆*0*) genetic background. Knockout strains were generated by one-step gene replacement (Longtine *et al*., 1998). The *sch9*∆, *cyr1::mTn,* and *ras2*∆ mutants have been described previously (Fabrizio *et al*., 2001; Wei *et al*., 2008). Strains with STRE-LacZ reporter gene were generated by integrating into the URA3 locus the pMM2 plasmid which contains four tandem repeats of STRE motif from the HSP12 sequence (−221 to −241) fused with the LacZ-coding sequence (Boy-Marcotte *et al*., 1998). The plasmid pCDV454 containing LacZ reporter under the control of a 37-bp SSA3-PDS region (−206 to −170) (Pedruzzi *et al*., 2000) was integrated into the URA3 locus of wild-type cells to generate PDS-LacZ gene reporter strain. The protocol of reporter gene activity measurement has been described elsewhere (Wei *et al*., 2008).

### Growth conditions

Yeast cells were grown in SDC containing 2% or 0.5% glucose supplemented with a 4-fold excess of tryptophan, leucine, histidine, and uracil to avoid possible lifespan modification due to auxotrophic deficiencies of the strains. Standard SDC medium without excess of essential amino acids was used for comparison. Day 3 wild-type cultures were centrifuged, and filtered supernatants were used to perform medium switch experiments.

### Chronological lifespan assay

Yeast chronological lifespan was measured as previously described (Fabrizio and Longo, 2003). Overnight SDC culture was diluted (OD 0.1) into fresh SDC medium (with 5:1 flask to culture volume) and incubated at 30 °C with shaking (200 rpm). 24 h later was considered as day 1. Every 48 h, properly diluted culture was plated onto YPD plates and then incubated at 30 °C for 2–3 days. Chronological lifespan was assessed by colony-forming units (CFUs) measurement. Day 3 CFUs were considered as the initial survival (100%) and used to determine the age-dependent mortality. Mean lifespan was calculated from curve fitting of the survival data with the statistical software Prism (GraphPad Software, Inc., La Jolla, CA, USA). For medium switch experiments, cells were grown in SDC for 3 days, washed twice with sterile distilled water, and suspended in MES buffer (40 mm, pH 3.7 or pH 6.0) with different carbon sources or other media as stated.

### Stress resistance assay

Heat shock resistance was measured by spotting serial dilutions (5-fold or 10-fold dilution) of cells removed from cell cultures onto YPD plates and incubating at 55 °C (heat-shocked) and at 30 °C (control) for 60–150 min. After the heat shock, plates were transferred to 30 °C and incubated for 2 days. For oxidative stress resistance assays, cells were diluted 10-fold in K-phosphate buffer (pH 6.0) and treated with 70–300 mm H_2_O_2_ for 30 min. Serial dilutions (5-fold or 10-fold) of untreated control and H_2_O_2_-treated cells were spotted onto YPD plates and incubated at 30 °C for 2–3 days.

### LacZ reporter gene assay

1 mL culture was collected, washed once with water, pelleted, and then stored at −80 °C. 100 μL of low-salt lysis buffer (50 mm Tris, pH 7.5, 0.1% (v/v) Triton X-100, 1X protease inhibitor, 100 mm NaCl, 2 mm EDTA, 2 mm EGTA, 50 mm NaF) and an equal volume of glass beads were added to the cell pellets. The mixture was vortexed at maximum speed for 45 s and then chilled on ice for 45 s. This was repeated five times. The mix was centrifuged at 14 000 rpm at 4 °C for 5 min and the supernatant was transferred to a clean tube. Protein concentration of the lysate was assayed with a BCA kit (Pierce). 55 μL of lysate (or appropriately diluted samples) was mixed with 85 μL of substrate solution (1.1 mg mL^−1^ ONPG in Z buffer: 60 mm Na_2_HPO_4_, 40 mm NaH_2_PO_4_, 10 mm KCl, 1 mm MgSO_4_, 50 mm 2-mercaptoethanol, pH 7.0). Absorbance at 420 nm was read every 5 min until 30 min after the reaction was started. LacZ activities were determined by fitting the A420/time data to that of serial-diluted recombinant β-galactosidase (Promega) and were further normalized with total protein.

### Acetic acid and ethanol measurements

Yeast chronological aging cultures were centrifuged, and supernatants were collected and frozen at −80 °C. Acetic acid and ethanol concentrations of the conditioned medium were determined by acetic acid and ethanol assay kits (R-biopham Cat. 10148261035 and 10176290035) following the manufacturer’s recommended protocol.

### Oxygen consumption measurement

Oxygen consumption of 2 mL culture was measured in a glass container magnetically stirred to avoid cell precipitation at 30 °C in a water bath using a Clark-type electrode. According to the manufacturer, the liquid culture was assumed to contain the same amount of oxygen as water equilibrated with 21% oxygen in 1 atmosphere pressure (5.02 μL mL^−1^). Oxygen level in the culture was automatically recorded every 5 s until a trace of straight line was obtained, which suggested that the oxygen consumption had reached a steady state. The amount of oxygen consumed was further normalized by CFU.

### Quantification of glycogen and trehalose

2.5–3 mL cell cultures was collected from indicated cultures at indicated time points. Cells were washed with water, pelleted, and stored at −80 °C until measurement. The cell pellet weights were measured at the time of collection for normalization purpose. Intracellular glycogen and trehalose contents were determined as previously described (Parrou & Francois, 1997). Briefly, cell pellets were resuspended in 250 μL of 0.25 m Na_2_CO_3_ and incubated at 95 °C with occasional stirring for 4 h. Subsequently, the suspension was buffered to pH 5.2 by adding 150 μL of 1 m acetic acid and 600 μL of 0.2 m NaAc (pH 5.2). For the trehalose content determination, half of the mixture was incubated overnight at 37 °C in the presence of 3 mm trehalase (Sigma, USA) under constant agitation. For the glycogen content determination, the second half was digested overnight at 57 °C with continuous shaking on a rotary shaker in the presence of 100 μg α-amyloglucosidase (Sigma-Aldrich, St. Louis, MO, USA). Finally, mixture was centrifuged and the supernatant was collected. Glucose released from trehalose and glycogen digestion was quantified using the Glucose Assay Kit (R-biopham, Cayman Chemical Company, Ann Arbor, MI, USA) following the manufacturer’s protocol.

## Supporting Information

**Fig. S1** (A, B) Cells were grown in 2% glucose SDC medium until day 2. **Fig. S2** (A, B) Wild-type (DBY746) growing in 2% glucose SDC with 4X or 1X HIS, URA, LEU, TRP, or 1X LEU with 4X IS, URA, TRP. **Fig. S3** (A, B) Wild-type and sch9Δ mutant cells grown in 2% glucose SDC until day 3 and then washed 3 times and switched to 40 mm MES buffer (pH 3.7) or buffer with physiological levels of ethanol (0.8%) or acetic acid (50 mm). **Fig. S4** (A) Chronological survival of wild-type cells in media limited in leucine.
