# Supplementary material for: Tor-Sch9 deficiency activates catabolism of the ketone body-like acetic acid to promote trehalose accumulation and longevity
Source: Aging Cell. 2014 Mar 20;13(3):457–67. doi: 10.1111/acel.12202 (PMC4032597; doi:10.1111/acel.12202)

Supplementary Figures:

Figure S1. (A, B) Cells were grown in 2% glucose SDC medium until day2. Then cells were equally split into two flasks and one culture was buffered with 0.1 M MES and NaOH to pH 6. Samples were collected 8, 24 and 48 hours after buffering (n=3). *, $p<0.05$. (C) The pH of the cultures during chronological aging. (D) Extracellular ethanol concentrations of wild type, *ady2Δ, pdc6Δ* and *acs1*Δ mutants.

Figure S2. (A, B) Wild type (DBY746) growing in 2% glucose SDC with 4X or 1X HIS, URA, LEU, TRP or 1X LEU with 4X HIS, URA, TRP. Extracellular acetate and ethanol concentration normalized to cell numbers (n=3). (C) The extracellular acetate concentration of BY4741 wild type cells grown in SDC media with 4X and 8X LEU (n=4). (D) Chronological survival of *tor1Δ and sch9Δ* mutant cells prototrophic for LEU2 gene in SDC –leucine media.

Figure S3. (A, B) Wild type and *sch9Δ* mutant cells grown in 2% glucose SDC until day 3 and then washed 3 times and switched to 40mM MES buffer (pH 3.7) or buffer with physiological levels of ethanol (0.8%) or acetic acid (50mM). Oxygen consumption were monitored 3 and 24 hours after the switch (N=4-8) (C) O_2 consumption of wild type (BY4741) and *sch9Δ* mutants during chronological aging (n=2-5). (D) The *sch9Δ* mutant is treated with NaCN (0.25mM) on day1, then every other day until day7. Acetic acid concentration in cultures was monitored every other day. (E, F) Extracellular acetate and ethanol in the cultures of *coq* mutants on days 3 and 7 (n=3)

Figure S4. (A) Chronological survival of wild type cells in media limited in leucine. (B) Chronological survival of *bat1Δ bat2Δ* double mutants in standard SDC media.

Figure S1

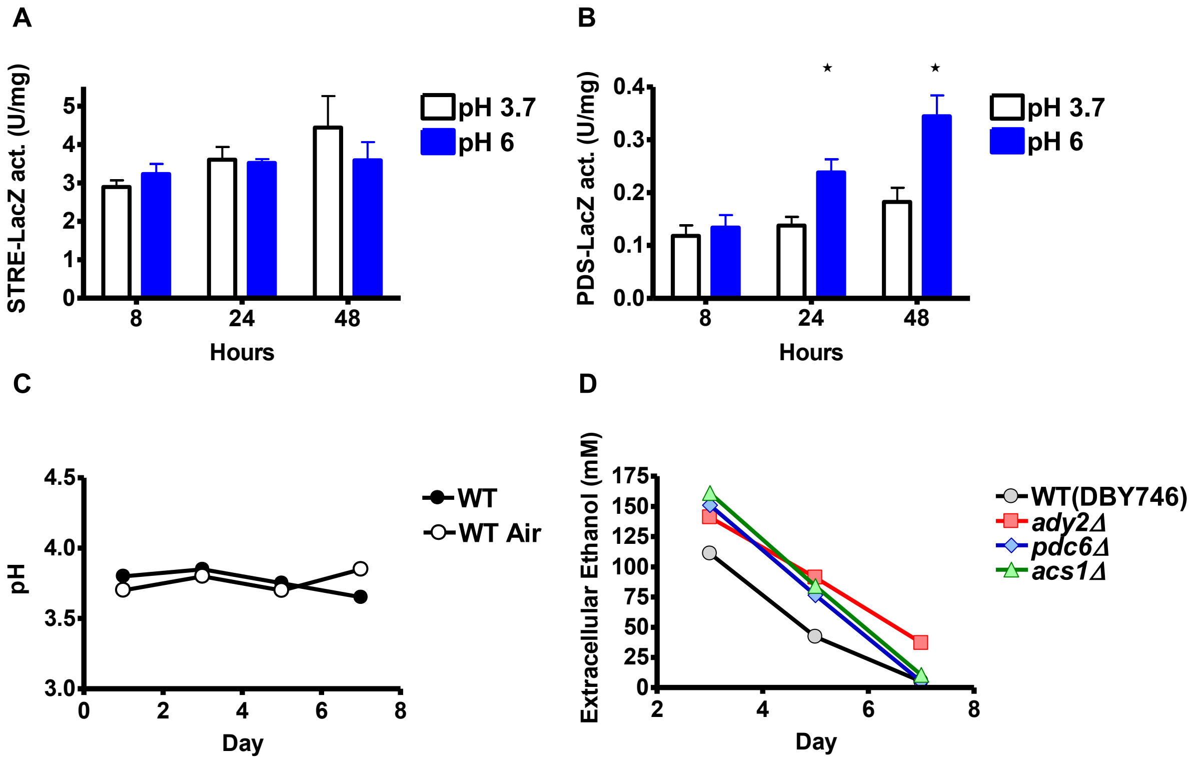
A
STRE-LacZ act. (U/mg)
0
1
2
3
4
5
8
24
48
Hours
pH 3.7
pH 6
B
PDS-LacZ act. (U/mg)
0.0
0.1
0.2
0.3
0.4
8
24
48
Hours
pH 3.7
pH 6
C
pH
3.0
3.5
4.0
4.5
0
2
4
6
8
Day
WT
WT Air
D
Extracellular Ethanol (mM)
0
25
50
75
100
125
150
175
2
4
6
8
Day
WT(DBY746)
ady2Δ
pdc6Δ
acs1Δ

Figure S2

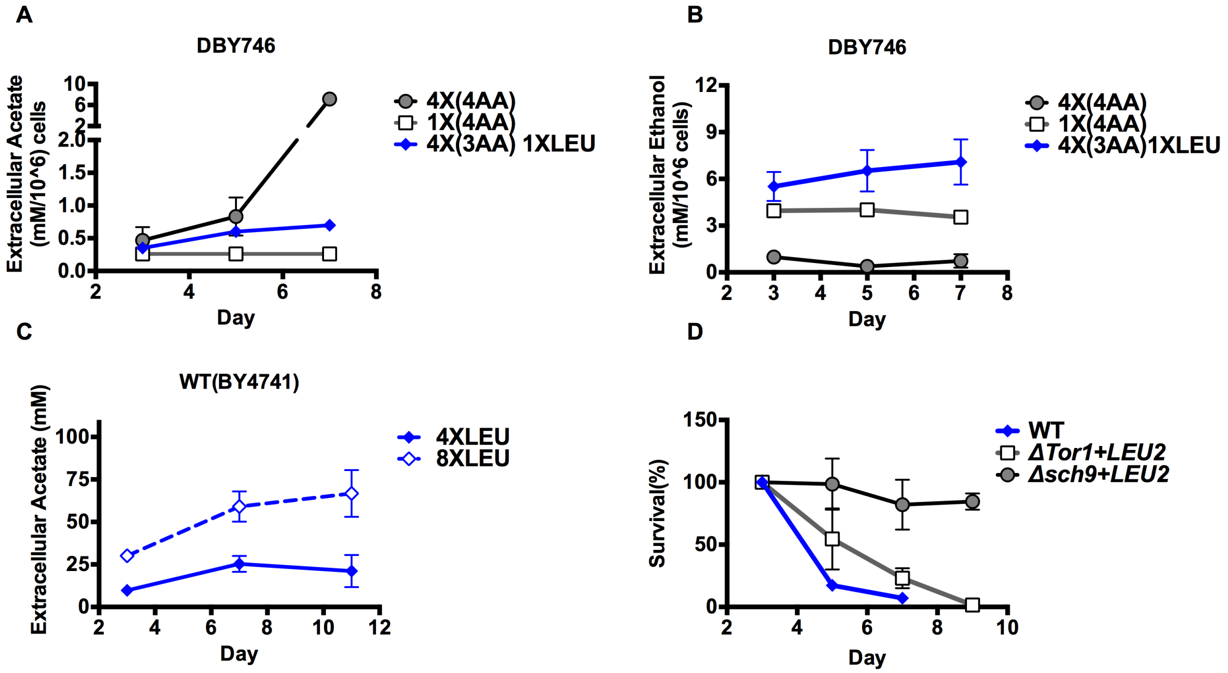
A
DBY746
Extracellular Acetate (mM/10^6) cells
Day
4X(4AA)
1X(4AA)
4X(3AA) 1XLEU
B
DBY746
Extracellular Ethanol (mM/10^6 cells)
Day
4X(4AA)
1X(4AA)
4X(3AA)1XLEU
C
WT(BY4741)
Extracellular Acetate (mM)
Day
4XLEU
8XLEU
D
Survival(%)
Day
WT
ΔTor1+LEU2
Δsch9+LEU2

Figure S3

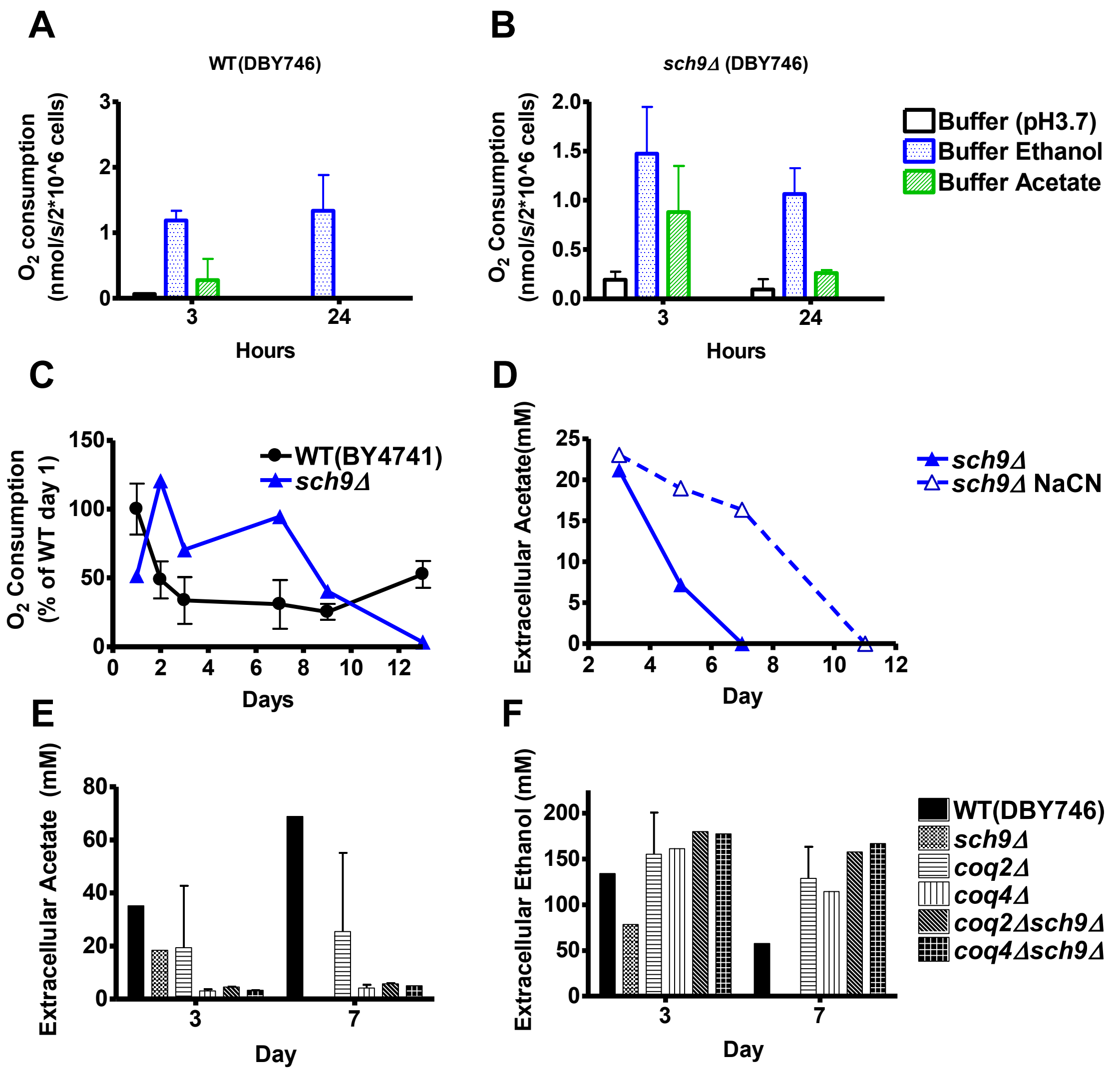

A
WT(DBY746)
O2 consumption (nmol/s/2*10^6 cells)
Hours
B
sch9Δ (DBY746)
O2 Consumption (nmol/s/2*10^6 cells)
Hours
Buffer (pH3.7)
Buffer Ethanol
Buffer Acetate
C
O2 Consumption (% of WT day 1)
WT(BY4741)
sch9Δ
Days
D
Extracellular Acetate(mM)
sch9Δ
sch9Δ NaCN
Day
E
Extracellular Acetate (mM)
Day
F
Extracellular Ethanol (mM)
Day
WT(DBY746)
sch9Δ
coq2Δ
coq4Δ
coq2Δsch9Δ
coq4Δsch9Δ

Figure S4

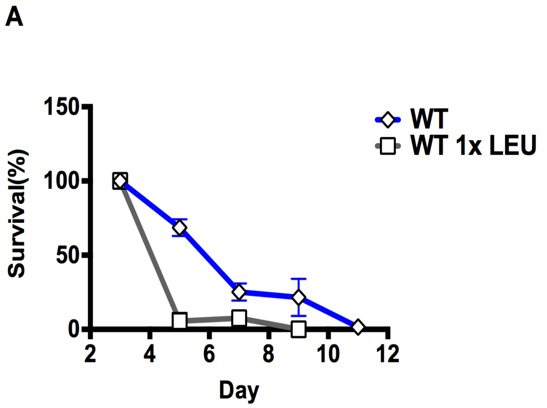

Supplement: Supplementary file 1 — Fig. S1 (A, B) Cells were grown in 2% glucose SDC medium until day 2. Fig. S2 (A, B) Wild-type (DBY746) growing in 2% glucose SDC with 4X or 1X HIS, URA, LEU, TRP, or 1X LEU with 4X IS, URA, TRP. Fig. S3 (A, B) Wild-type and sch9Δ mutant cells grown in 2% glucose SDC until day 3 and then washed 3 times and switched to 40 mm MES buffer (pH 3.7) or buffer with physiological levels of ethanol (0.8%) or acetic acid (50 mm). Fig. S4 (A) Chronological survival of wild-type cells in media limited in leucine. [file acel0013-0457-sd1.pdf]
